# Treatment patterns and clinical outcomes of resectable clinical stage III non‐small cell lung cancer in a Japanese real‐world setting: Surgery cohort analysis of the SOLUTION study

**DOI:** 10.1111/1759-7714.15305

**Published:** 2024-05-29

**Authors:** Masahiro Tsuboi, Haruyasu Murakami, Hideyuki Harada, Tomotaka Sobue, Tomohiro Kato, Shinji Atagi, Takaaki Tokito, Tadashi Mio, Hirofumi Adachi, Toshiyuki Kozuki, Takashi Sone, Masahiro Seike, Shinichi Toyooka, Hiroshi Kitagawa, Ryo Koto, Satoshi Yamazaki, Hidehito Horinouchi

**Affiliations:** ^1^ Department of Thoracic Surgery National Cancer Center Hospital East Chiba Japan; ^2^ Division of Thoracic Oncology Shizuoka Cancer Center Shizuoka Japan; ^3^ Division of Radiation Therapy Shizuoka Cancer Center Shizuoka Japan; ^4^ Division of Environmental Medicine and Population Sciences Graduate School of Medicine, Osaka University Osaka Japan; ^5^ Department of Respiratory Medicine National Hospital Organization Himeji Medical Center Hyogo Japan; ^6^ Department of Thoracic Oncology National Hospital Organization Kinki‐Chuo Chest Medical Center Osaka Japan; ^7^ Division of Respirology, Neurology, and Rheumatology, Department of Internal Medicine Kurume University Hospital Fukuoka Japan; ^8^ Department of Respiratory Medicine National Hospital Organization Kyoto Medical Center Kyoto Japan; ^9^ Department of Thoracic Surgery National Hospital Organization Hokkaido Cancer Center Hokkaido Japan; ^10^ Department of Thoracic Oncology and Medicine National Hospital Organization Shikoku Cancer Center Ehime Japan; ^11^ Department of Respiratory Medicine Kanazawa University Hospital Ishikawa Japan; ^12^ Department of Pulmonary Medicine and Oncology Graduate School of Medicine, Nippon Medical School Tokyo Japan; ^13^ Department of General Thoracic Surgery and Breast and Endocrinological Surgery, Okayama University Graduate School of Medicine Dentistry and Pharmaceutical Sciences Okayama Japan; ^14^ Medical Department AstraZeneca K.K. Osaka Japan; ^15^ Department of Thoracic Oncology National Cancer Center Hospital Tokyo Japan

**Keywords:** disease‐free survival, perioperative therapy, resectability, stage III non‐small cell lung cancer, surgery

## Abstract

**Background:**

To elucidate the treatment and surgery outcomes with or without perioperative therapies in Japanese patients with clinical stage III non‐small cell lung cancer (NSCLC) in real‐world settings.

**Methods:**

We performed subset analyses of the SOLUTION study, a multicenter, noninterventional, observational study of Japanese patients diagnosed with clinical stage III NSCLC, for those who started first‐line treatment (surgery±perioperative therapy) between January 2013 and December 2014 (study registration: UMIN000031385). Follow‐up data were obtained using medical records from diagnosis to March 1, 2018.

**Results:**

Of 149 eligible patients, 67 underwent surgery alone (median age 71 years) and 82 underwent surgery+perioperative therapy (median age 63 years). Lung resection was performed in 137 patients and the others underwent exploratory thoracotomy or other procedures. Perioperative therapies included adjuvant therapy only (*n* = 41), neoadjuvant therapy only (*n* = 24), and neoadjuvant+adjuvant therapy (*n* = 17). The median overall survival (OS) and 3‐year OS rate were 29.3 months and 44.0%, respectively, in patients who underwent surgery alone, and not reached and 61.1%, respectively, in patients who underwent surgery+perioperative therapy. The 3‐year progression‐free survival (PFS) and disease‐free survival (DFS) rates were 42.4% and 47.1%, respectively, in patients who underwent surgery+perioperative therapy and 28.5% and 28.9%, respectively, in patients who underwent surgery alone. In multivariable Cox regression, perioperative therapy was associated with improved OS (hazard ratio [95% confidence interval] 0.49 [0.29–0.81]), PFS (0.62 [0.39–0.96]), and DFS (0.62 [0.39–0.97]) versus surgery alone.

**Conclusions:**

Our study suggested that perioperative therapy may be associated with better survival among patients undergoing surgical treatment of clinical stage III NSCLC.

## Graphical abstract

We investigated the characteristics and outcomes of Japanese patients who underwent surgery with or without perioperative therapies for clinical stage III non‐small cell lung cancer (NSCLC) in real‐world settings. We found that surgery+perioperative therapy provided more favorable survival outcomes than surgery only, and that this treatment strategy should be considered for patients who would be expected to tolerate it. These real‐world data will be useful as a benchmark for future therapies.

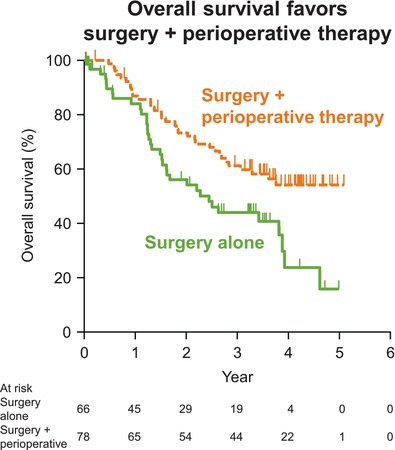

## INTRODUCTION

Surgical resection is the mainstay treatment for stage I–II non‐small cell lung cancer (NSCLC) offering a good chance of cure.^1^, ^2^ The Japan Lung Cancer Society clinical guidelines recommend surgery for patients with resectable stage IIIA NSCLC. Recommended surgical procedures include lobectomy, lymph node dissection, and combined resection of chest wall or pericardium, if involved.^2^ Patients with resectable stage IIIA NSCLC also benefit from perioperative (neoadjuvant and/or adjuvant) therapies.^3^, ^4^ However, they may not be feasible in all patients, particularly in older patients with a relatively poor performance status.^5^

A previous Japanese registry described the characteristics and outcomes of surgically treated patients with stage I–IV NSCLC.^6^ In that study, 28.6% (5188/18127) of patients who underwent R0 or R1 resection received adjuvant chemotherapy, of which 14.9% received platinum doublet chemotherapy. However, the actual treatments of patients with resectable clinical stage III NSCLC in Japan remain unclear. Furthermore, there may be some situations where the clinical guidelines cannot be followed entirely. The SOLUTION study investigated the treatment realities in 744 patients with clinical stage III NSCLC in a real‐world setting.^7^ Here, we describe the patient and treatment characteristics, use of perioperative therapies, and outcomes of Japanese patients with clinical stage III NSCLC who underwent surgical resection. We used real‐world data from a period when chemotherapy was the only adjuvant/neoadjuvant therapy used in the perioperative period. We expect the results will serve as benchmark data for the future, when new therapies such as immune checkpoint inhibitors and molecular‐targeted drugs are available.

## METHODS

### Overview and ethics

As previously described,^7^ this multicenter, noninterventional, observational study was registered at the University Hospital Medical Information Network Clinical Trials Registry (UMIN000031385). The study adhered to the Japanese Ethical Guidelines for Medical and Health Research Involving Human Subjects,^8^ which incorporate the ethical principles defined in the Declaration of Helsinki. A total of 11 sites participated with approval from the ethical review boards at each site. The 11 sites are large clinical centers, distributed widely across Japan, as representative hospitals of lung cancer surgery in each region, and often have their own policies on cancer treatment.

### Patients

The clinicians reviewed the medical records of their patients who were diagnosed with clinical stage III NSCLC at ≥20 years old and who started first‐line treatment (chemotherapy, chemoradiotherapy [CRT], radiotherapy, or surgery) between January 2013 and December 2014. Their medical records were used to follow‐up the patients until March 1, 2018 using case report forms. Informed consent was obtained from surviving patients prior to database registration. As previously described,^7^ patients who died or moved from the participating site during the follow‐up period could be registered, providing the study information was recorded and the patient or legal representative had an opportunity to opt out of the study, in accordance with the requirements stipulated by the site's ethical review board. In this report, we focused on patients with clinical stage III NSCLC who underwent surgery with or without perioperative adjuvant/neoadjuvant therapy (Figure S1a). NSCLC was staged according to the American Joint Committee on Cancer Staging Manual, version 7, based on the pathological information available in the patients' medical records. Other inclusion/exclusion criteria are described in the prior report.^7^

### Data collection, endpoints, and analyses

The data included patient characteristics at the time of NSCLC diagnosis, details of the surgical procedure and perioperative treatment (if used), overall survival (OS), progression‐free survival (PFS), disease‐free survival (DFS), local progression, and distant metastasis. We also recorded deaths occurring within 30 or 90 days after surgery as an indicator of surgery‐related complications. The extent of lymph node dissection was classified according to the Japan Lung Cancer Society General Rule for Clinical and Pathological Record of Lung Cancer.^9^ The case report forms included the option “not evaluated” for disease staging and “yes” or “no” for surgical methods; additional information was not collected for these responses. The reasons and indications for performing or not performing specific surgical procedures were not recorded.

We performed descriptive analyses of patient characteristics, treatments, and deaths within 30 or 90 days, as an index of postoperative mortality, for all patients who underwent surgery (±perioperative therapy) and in the following subsets: patients who underwent surgery+perioperative therapy and patients who underwent surgery alone. We also estimated survival outcomes (OS, PFS, DFS, and distant metastasis) with Kaplan–Meier survival analysis for all patients and in the following subsets by treatment strategy: patients who received neoadjuvant+adjuvant therapy, patients who received neoadjuvant therapy, patients who received adjuvant therapy, and patients who underwent surgery alone. OS and PFS were determined for all evaluable patients. PFS included local or distant progression. DFS was defined as the time from surgery to disease recurrence in patients who underwent R0 resection. Patients were censored from March 1, 2018 onwards, at the last follow‐up (for OS), and/or the start of second‐line treatment (for PFS and DFS).

Potential covariates for OS, PFS, and DFS were evaluated in post‐hoc analyses using multivariable Cox proportional hazards models to account for potential confounding patient factors on survival outcomes. These analyses included the following covariates: use of perioperative therapy (no vs. yes), age (≥75 vs. <75 years), sex, smoking history (never smoker vs. current/past smoker), Eastern Cooperative Oncology Group performance status (0–1 vs. ≥2), histological type (squamous cell carcinoma [SCC] vs. other histological types [non‐SCC]), presence of chronic obstructive pulmonary disease (no vs. yes), and presence of interstitial pneumonia (no vs. yes). These characteristics were selected based on our knowledge and with reference to prior studies.^10^, ^11^, ^12^ For these analyses, the hazard ratios (HR) and 95% confidence intervals (CI) were calculated.

All analyses were descriptive and no formal statistical comparisons were made among the patient groups. Statistical analyses were performed using SAS System Release 9.2 or higher (SAS Inc., Cary, NC, USA).

The sponsor was involved in data analysis and interpretation; the authors retained full editorial control of the manuscript.

## RESULTS

### Patients

Of 744 registered patients, surgery (with or without perioperative therapy) was scheduled for 149 patients. A total of 67 patients underwent surgery alone and 82 underwent surgery+perioperative therapy (Figure S1b). Among the 149 patients, surgery was not performed as planned in five patients due to disease progression, adverse events following neoadjuvant therapy, or other reasons. One patient who underwent surgery alone and four patients who underwent surgery+perioperative therapy were included in the analyses for each cohort based on their intended treatments.

The median age of the overall surgery cohort (Table 1) was 67 years (range 34–84 years) and 83.9% of patients were male. Most patients had a performance status of 0 (72.5%) and nearly all had clinical stage IIIA NSCLC (96.0%). The p‐stage ranged from stage IA (3.4%) to IIIB (5.4%), but it was not evaluated in 23.5% of patients. Most of the patients had at least one comorbidity (>70%), such as chronic obstructive pulmonary disease (27.5%) and interstitial pneumonia (10.1%).

**TABLE 1:** Patient characteristics.

Characteristic	Overall surgery cohort (*n* = 149)	Surgery alone (*n* = 67)	Surgery + perioperative therapy (*n* = 82)
Age, years, median (range)	67 (34–84)	71 (49–84)	63 (34–78)
Age group (years)			
≥20–<65	63 (42.3)	15 (22.4)	48 (58.5)
≥65–<75	59 (39.6)	29 (43.3)	30 (36.6)
≥75	27 (18.1)	23 (34.3)	4 (4.9)
Sex			
Male	125 (83.9)	55 (82.1)	70 (85.4)
Female	24 (16.1)	12 (17.9)	12 (14.6)
Smoking history			
Current smoker	62 (41.6)	21 (31.3)	41 (50.0)
Past smoker	77 (51.7)	41 (61.2)	36 (43.9)
Never smoker	10 (6.7)	5 (7.5)	5 (6.1)
ECOG PS			
0	108 (72.5)	49 (73.1)	59 (72.0)
1	35 (23.5)	14 (20.9)	21 (25.6)
2	3 (2.0)	2 (3.0)	1 (1.2)
3/4	0	0	0
c‐stage			
Stage IIIA	143 (96.0)	66 (98.5)	77 (93.9)
T3‐N1	26 (17.4)	15 (22.4)	11 (13.4)
T4‐N1	6 (4.0)	4 (6.0)	2 (2.4)
T4‐N0	17 (11.4)	9 (13.4)	8 (9.8)
T1a‐N2	10 (6.7)	3 (4.5)	7 (8.5)
T1b‐N2	19 (12.8)	5 (7.5)	14 (17.1)
T2a‐N2	35 (23.5)	11 (16.4)	24 (29.3)
T2b‐N2	11 (7.4)	7 (10.4)	4 (4.9)
T3‐N2	19 (12.8)	12 (17.9)	7 (8.5)
Stage IIIB	6 (4.0)	1 (1.5)	5 (6.1)
p‐stage			
Stage IA	5 (3.4)	2 (3.0)	3 (3.7)
Stage IB	7 (4.7)	3 (4.5)	4 (4.9)
Stage IIA	3 (2.0)	2 (3.0)	1 (1.2)
Stage IIB	16 (10.7)	8 (11.9)	8 (9.8)
Stage IIIA	75 (50.3)	28 (41.8)	47 (57.3)
Stage IIIB	8 (5.4)	3 (4.5)	5 (6.1)
Not evaluated	35 (23.5)	21 (31.3)	14 (17.1)
Assessment of p‐TNM stage			
N2^a^, *n*	97	38	59
Performed	79 (81.4)	30 (78.9)	49 (83.1)
Not performed	18 (18.6)	8 (21.1)	10 (16.9)
Histological type			
Adenocarcinoma	70 (47.0)	25 (37.3)	45 (54.9)
SCC	61 (40.9)	32 (47.8)	29 (35.4)
Neuroendocrine tumor (NSCLC)	4 (2.7)	2 (3.0)	2 (2.4)
Other	14 (9.4)	8 (11.9)	6 (7.3)
Primary lesion location			
Right upper lobe	59 (39.6)	26 (38.8)	33 (40.2)
Right middle lobe	9 (6.0)	2 (3.0)	7 (8.5)
Right lower lobe	28 (18.8)	17 (25.4)	11 (13.4)
Left upper lobe	41 (27.5)	14 (20.9)	27 (32.9)
Left lower lobe	13 (8.7)	9 (13.4)	4 (4.9)
Unknown	1 (0.7)	0	1 (1.2)
Comorbidities			
COPD	41 (27.5)	18 (26.9)	23 (28.0)
Autoimmune disease	7 (4.7)	4 (6.0)	3 (3.7)
Interstitial pneumonia	15 (10.1)	9 (13.4)	6 (7.3)
IPF^b^	6 (40.0)	4 (44.4)	2 (33.3)
Non‐IPF^b^	4 (26.7)	3 (33.3)	1 (16.7)
Unknown^b^	5 (33.3)	2 (22.2)	3 (50.0)
Others	108 (72.5)	48 (71.6)	60 (73.2)

*Note*: Values are *n* (%) unless otherwise stated.

Abbreviations: COPD, chronic obstructive pulmonary disease; ECOG PS, Eastern Cooperative Oncology Group performance status; IPF, idiopathic pulmonary fibrosis; NSCLC, non‐small cell lung cancer; SCC, squamous cell carcinoma; TNM, tumor‐node‐metastasis.

^a^ Percentages were calculated using the number of patients with N2 disease as the denominator.

^b^ Percentages were calculated using the number of patients with interstitial pneumonia as the denominator.

The median age was 71 years in patients who underwent surgery alone and 63 years in patients who underwent surgery+perioperative therapy. While the proportion of the patients with chronic obstructive pulmonary disease was comparable between those who underwent surgery alone and those who underwent surgery+perioperative therapy (26.9% vs. 28.0%), the proportion of patients with interstitial pneumonia was numerically higher in those who underwent surgery (13.4% vs. 7.3%). Stage IIIB NSCLC was found in 1.5% of patients who underwent surgery alone and in 6.1% of patients who underwent surgery+perioperative therapy. P‐stage was IIIA in 41.8% of patients in the surgery alone group and 57.3% in the surgery+perioperative therapy group. The N2 node status was pathologically assessed in 78.9% of patients who underwent surgery alone and in 83.1% who underwent surgery+perioperative therapy.

Adenocarcinoma and SCC were the most common types of NSCLC in this cohort (47.0% and 40.9%, respectively). Adenocarcinoma was less frequent than SCC (37.3% and 47.8%, respectively) in patients who underwent surgery alone. Adenocarcinoma was more frequent than SCC in patients who underwent surgery+perioperative therapy (54.9% and 35.4%, respectively).

Epidermal growth factor receptor (*EGFR*) gene mutation tests were performed in 34.9% (52/149) of patients prior to first‐line treatment, including 38 patients with adenocarcinoma, nine with SCC, and five with other histological types. Six of the tested patients were positive for *EGFR* mutations; all of them had adenocarcinoma and underwent surgery+perioperative therapy (Table S1).

### Surgical procedures and lymph node dissection

The surgical procedures and extent of lymph node dissection are presented in Table 2. Overall, 137 (91.9%) patients underwent lung resection, of which 89.1% underwent lobectomy including bilobectomy. The other patients underwent exploratory thoracotomy or other procedures; the detailed information about the procedures was unavailable for these patients. Adjunctive surgical procedures were performed in 34 patients. Lymph node dissection was performed in 109 patients, most of whom underwent systematic lymph node dissection.

**TABLE 2:** Surgical procedures and extent of lymph node dissection.

Procedure	Overall surgery cohort (*n* = 149)	Surgery alone (*n* = 67)	Surgery + perioperative therapy (*n* = 82)
Operative procedures			
Lung resection	137 (91.9)	61 (91.0)	76 (92.7)
Lymph node dissection	109 (73.2)	45 (67.2)	64 (78.0)
Adjunctive procedure	34 (22.8)	15 (22.4)	19 (23.2)
Exploratory thoracotomy	9 (6.0)	5 (7.5)	4 (4.9)
Type of lung resection^a^, *n*	137	61	76
Lobectomy (including bilobectomy)	122 (89.1)	52 (85.2)	70 (92.1)
Pneumonectomy	9 (6.6)	5 (8.2)	4 (5.3)
Wedge resection	4 (2.9)	3 (4.9)	1 (1.3)
Segmentectomy	3 (2.2)	1 (1.6)	2 (2.6)
Adjunctive procedures^a^, *n*	34	15	19
Combined resection of adjacent organs	18 (52.9)	7 (46.7)	11 (57.9)
Tracheobronchoplasty	7 (20.6)	3 (20.0)	4 (21.1)
Angioplasty	4 (11.8)	2 (13.3)	2 (10.5)
Extrapleural pneumonectomy	0	0	0
Other adjunctive procedures	8 (23.5)	3 (20.0)	5 (26.3)
Lymph node dissection^a^, *n*	109	45	64
No lymph node dissection	1 (0.9)	1 (2.2)	0
Sampling lymph node dissection^b^	4 (3.7)	4 (8.9)	0
Lobe‐specific lymph node dissection^c^	31 (28.4)	9 (20.0)	22 (34.4)
Systematic lymph node dissection^d^	71 (65.1)	30 (66.7)	41 (64.1)
Extended lymph node dissection^e^	1 (0.9)	0	1 (1.6)
Unknown	1 (0.9)	1 (2.2)	0

*Note*: Values are *n* (%) unless otherwise stated. Some patients underwent multiple types of procedures. If the same patient underwent the same procedure multiple times, the patient was counted once.

^a^ Percentages were calculated using the number of patients who underwent the indicated procedure type as the denominator.

^b^ Includes ND1a‐1b dissection.

^c^ Includes ND2a‐1 dissection.

^d^ Includes ND2a‐2 to ND2b dissection.

^e^ Includes ND3a‐3γ dissection.

### Perioperative regimens

A total of 82 patients received perioperative therapies: adjuvant therapy alone in 41, neoadjuvant therapy alone in 24, and neoadjuvant+adjuvant therapy in 17 (Table S2). Among patients who received neoadjuvant+adjuvant therapy, the most common neoadjuvant regimen was CRT with cisplatin+docetaxel, while the most common adjuvant regimen was chemotherapy with cisplatin+docetaxel. Among patients who received neoadjuvant therapy alone, the predominant regimen was CRT comprising cisplatin+docetaxel. Among patients who received adjuvant therapy, most received chemotherapy alone, with cisplatin+vinorelbine being the most frequently prescribed regimen. The median interval between surgery and the start of adjuvant therapy was 50 days (range, 25–80 days; reported for 54/58 patients who received adjuvant therapy) (data not shown).

### Overall survival and disease progression

The median follow‐up of the overall surgery cohort was 31.4 months. One patient died within 30 days (0.7%) and two died within 90 days (1.3%) from surgery; these patients underwent surgery alone (Table S3).

**FIGURE 1:**
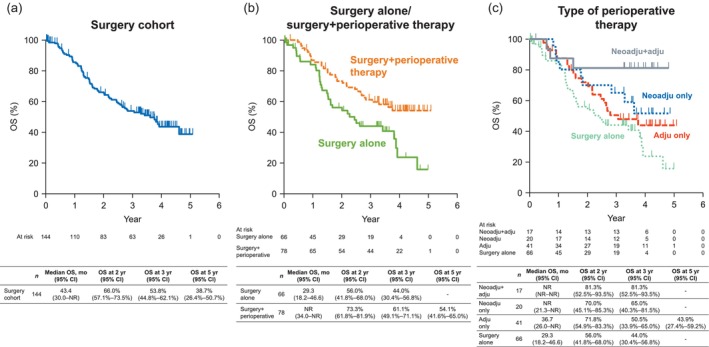
Overall survival in (a) the overall surgery cohort, (b) patients who underwent surgery alone or surgery+perioperative therapy, and (c) according to the type of perioperative therapy. adju, adjuvant therapy; CI, confidence interval; mo, month; neoadju, neoadjuvant therapy; NR, not reached; OS, overall survival; yr, year.

The median OS for the overall surgery cohort was 43.4 months and the 3‐year OS rate was 53.8% (Figure 1a). The median OS and 3‐year OS rate were 29.3 months and 44.0%, respectively, in patients who underwent surgery alone, and not reached and 61.1%, respectively, in patients who underwent surgery+perioperative therapy (Figure 1b). The 3‐year OS rate was 81.3% in patients who received neoadjuvant+adjuvant therapy, 65.0% in patients who received neoadjuvant therapy only, 50.5% in patients who received adjuvant therapy only, and 44.0% in patients who underwent surgery alone (Figure 1c).

**FIGURE 2:**
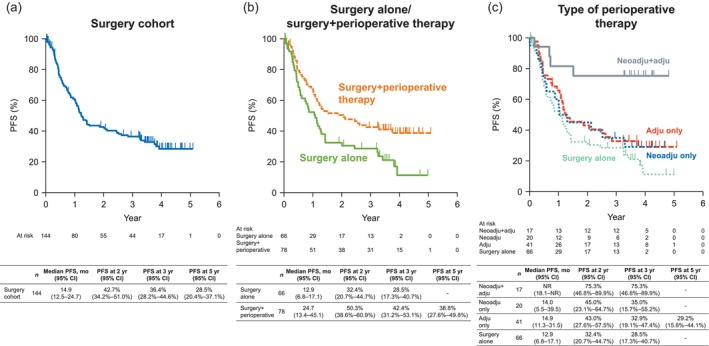
Progression‐free survival in (a) the overall surgery cohort, (b) patients who underwent surgery alone or surgery+perioperative therapy, and (c) according to the type of perioperative therapy. adju, adjuvant therapy; CI, confidence interval; mo, month; neoadju, neoadjuvant therapy; NR, not reached; PFS, progression‐free survival; yr, year.

In terms of PFS, the median value was 14.9 months and the 3‐year PFS rate was 36.4% in the overall surgery cohort (Figure 2a). In patients who underwent surgery alone, the median PFS and 3‐year PFS rates were 12.9 months and 28.5%, respectively. The corresponding values in patients who underwent surgery+perioperative therapy were 24.7 months and 42.4% (Figure 2b). The 3‐year PFS rate was 75.3% in patients who received neoadjuvant+adjuvant therapy, 35.0% in patients who received neoadjuvant therapy only, 32.9% in patients who received adjuvant therapy only, and 28.5% in patients who underwent surgery alone (Figure 2c). The median PFS was not reached, 14.0, 14.9, and 12.9 months, respectively.

The findings were similar for DFS. The median DFS was 16.7 months and the 3‐year DFS rate was 40.2% in the overall surgery cohort (Figure S2a). The corresponding values were 12.9 months and 28.9%, respectively, in patients who underwent surgery alone and were 31.5 months and 47.1%, respectively, in patients who underwent surgery+perioperative therapy (Figure S2b). The 3‐year DFS was 80.4% in patients who received neoadjuvant+adjuvant therapy, 38.9% in patients who received neoadjuvant therapy only, 35.9% in patients who received adjuvant therapy only, and 28.9% in patients who underwent surgery alone (Figure S2c). The median DFS was not reached, 20.5, 22.4, and 12.9 months, respectively.

A total of 78 patients experienced local progression and/or distant metastasis during the follow‐up period, of which 17 experienced local progression, 41 experienced distant metastasis, and 20 experienced both local progression and distant metastasis (Table 3). Local progression alone occurred in 27.8% of patients who underwent surgery alone and 16.7% of patients who underwent surgery+perioperative therapy. The proportion of patients who experienced local progression and distant metastasis was 19.4% in those who underwent surgery and 31.0% in those who underwent surgery+perioperative therapy. In the overall surgery cohort, distant metastases were most frequently found in the brain, bones, and lungs (23.0%, 21.3%, and 19.7%, respectively). Brain metastases were detected in 34.3% of patients who underwent surgery+perioperative therapy and in 7.7% of patients who underwent surgery alone. Bone, lung, and pleural metastases were found in 30.8%, 23.1%, and 23.1%, respectively, of patients who underwent surgery alone. The corresponding values for patients who underwent surgery+perioperative therapy were 14.3%, 17.1%, and 5.7%.

**TABLE 3:** Local progression and metastasis.

Progression/metastatic status	Overall surgery cohort (*n* = 149)	Surgery alone (*n* = 67)	Surgery + perioperative therapy (*n* = 82)
Patients with progression/metastasis	78 (52.3)	36 (53.7)	42 (51.2)
Only local progression^a^	17 (21.8)	10 (27.8)	7 (16.7)
Only distant metastasis^a^	41 (52.6)	19 (52.8)	22 (52.4)
Local progression and distant metastasis^a^	20 (25.6)	7 (19.4)	13 (31.0)
Unknown	0	0	0
Patients with distant metastases	61	26	35
Affected site^b^			
Brain	14 (23.0)	2 (7.7)	12 (34.3)
Bone	13 (21.3)	8 (30.8)	5 (14.3)
Lung	12 (19.7)	6 (23.1)	6 (17.1)
Pleura	8 (13.1)	6 (23.1)	2 (5.7)
Distant lymph nodes	8 (13.1)	4 (15.4)	4 (11.4)
Liver	8 (13.1)	4 (15.4)	4 (11.4)
Malignant pleural effusion	4 (6.6)	2 (7.7)	2 (5.7)
Adrenal gland	4 (6.6)	2 (7.7)	2 (5.7)
Peritoneum	1 (1.6)	0	1 (2.9)
Skin	1 (1.6)	1 (3.8)	0
Marrow	0	0	0
Other sites	5 (8.2)	3 (11.5)	2 (5.7)

*Note*: Values are *n* (%) of patients.

^a^ Percentages were calculated using the number of patients with progression/metastasis as the denominator.

^b^ Percentages were calculated using the number of patients with distant metastasis as the denominator.

As shown in Figure S3, the cumulative incidence of distant metastasis increased rapidly, reaching a plateau of about 55% by 2 years in patients who underwent surgery alone. In patients who underwent surgery+perioperative therapy, this plateau was reached at about 4–5 years.

**FIGURE 3:**
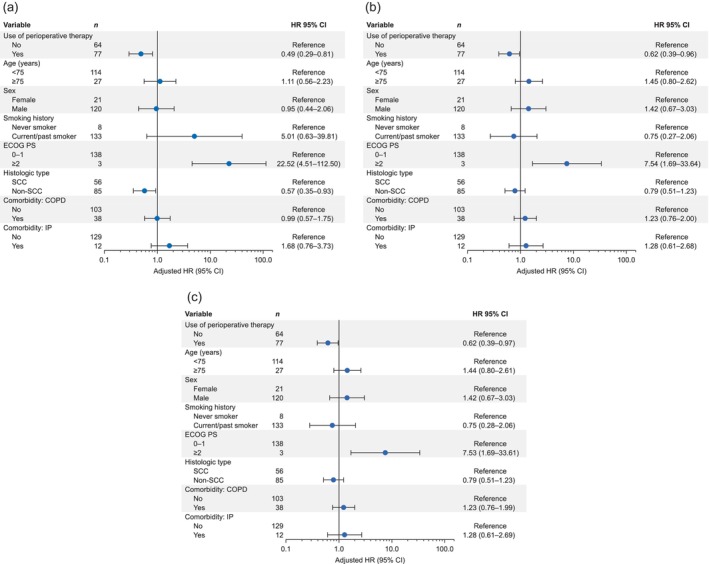
Multivariable Cox proportional hazards regression analysis of (a) overall survival, (b) progression‐free survival, and (c) disease‐free survival. CI, confidence interval; COPD, chronic obstructive pulmonary disease; ECOG PS, Eastern Cooperative Oncology Group performance status; HR, hazard ratio; IP, interstitial pneumonia; SCC, squamous cell carcinoma.

In multivariable Cox regression, the use of perioperative therapy (HR: 0.49; 95% CI: 0.29–0.81) and non‐SCC (HR: 0.57; 95% CI: 0.35–0.93) were independently associated with better OS (Figure 3a). Perioperative therapy was also independently associated with PFS (HR: 0.62; 95% CI: 0.39–0.96; Figure 3b) and DFS (HR: 0.62; 95% CI: 0.39–0.97; Figure 3c).

## DISCUSSION

This cohort study revealed the characteristics and treatment outcomes of patients with stage III resectable NSCLC who underwent surgery alone or surgery+perioperative therapy in actual clinical practice in Japan. Notable findings of this study include a high proportion of elderly patients (aged ≥65 years) and that most patients aged ≥75 years underwent surgery alone. Generally, elderly patients poorly tolerate aggressive therapy because of their poor performance status, comorbidities, and deterioration of organ function, and they are at increased risk of peri‐/postoperative complications.^13^, ^14^ In the present study, the performance status was numerically similar between patients who underwent surgery alone, in which the majority were elderly, and patients who underwent surgery and perioperative therapy, which mostly comprised younger patients. Based on this finding, it can be assumed that the use of perioperative chemotherapy was avoided in elderly patients due to the presence of comorbidities, especially interstitial pneumonia or age‐related physical or organ dysfunction, without impacting on their performance status, or because they were regarded as unsuitable for perioperative therapy due to their age. Additionally, current guidelines are limited to cisplatin combination chemotherapy as postoperative adjuvant chemotherapy,^2^, ^15^ which potentially limited the number of elderly patients who could receive this treatment. Although our descriptive analyses revealed a very low rate of perioperative therapy in elderly patients (4.9% of patients who underwent perioperative therapy were ≥75 years old), very few reports have focused on patients with stage III NSCLC, for which there is a huge range of treatment options. Hence, a large cohort study will be needed to test our hypothesis that comorbidities and other age‐related factors contribute to decisions surrounding the use of perioperative therapy in older patients, particularly the low frequency of use in these patients.

Regarding the surgical procedures performed in this cohort of patients with stage III NSCLC, lobectomy was the preferred type of lung resection (89.1% of patients), followed by pneumonectomy (6.6%). In prior Japanese annual reports, lobectomy was performed in 74.2% of patients with stage I–IV NSCLC in 2013 and in 72.4% of patients in 2014, whereas pneumonectomy was performed in 1.5% and 1.4% of patients, respectively.^16^, ^17^ Furthermore, in another Japanese study, the frequency of pneumonectomy for patients with cN2/pN2 stage IIIA NSCLC decreased from 19.8% in 1994 to 10.6% in 2004.^18^ In contrast, among patients who underwent surgical treatment of stage III NSCLC in a Canadian study, 61% underwent lobectomy and 33% underwent pneumonectomy.^19^ In a study of patients with stage IIIA NSCLC in the Netherlands, 77% of patients underwent lobectomy and 15% of patients underwent pneumonectomy.^20^ These data suggest that the frequency of pneumonectomy for stage III NSCLC may be lower in Japan than in other countries. Possible reasons for the lower frequency of pneumonectomy may include advances in bronchoplasty and angioplasty,^21^ improvements in other surgical procedures that may avoid the need for pneumonectomy,^22^ and the decreasing proportion of patients with SCC (33.0% in 1994 to 22.3% in 2004) and increasing proportion of patients with adenocarcinoma (from 55.7% in 1994 to 67.9% in 2004) over time.^23^

The European Organization for Research and Treatment of Cancer suggested that surgery should not be denied based on the patient's age alone, and adjuvant chemotherapy is associated with a survival benefit in elderly patients^24^ but the risk of chemotherapy‐induced toxicity after surgical resection should be taken into account in such patients. Among 82 patients who underwent perioperative therapy, most (70.7%) received adjuvant therapy, predominantly platinum‐based regimens consistent with the Japanese guidelines. Carboplatin was also used in some patients, mostly in elderly patients because of its known effectiveness in this population. In a Canadian study,^19^ cisplatin‐based regimens were the predominant types of chemotherapy in patients who underwent surgery/multimodal surgery, which was consistent with our findings.

In recent years, EGFR tyrosine kinase inhibitors have shown promising results in patients with metastatic NSCLC.^25^, ^26^ In our study of stage III NSCLC, the *EGFR* mutation status was mainly assessed in patients with adenocarcinomas prior to first‐line therapy and only six (15.8%) of those patients were positive. However, we should acknowledge that *EGFR* testing (alongside testing for *ALK* fusion, *ROS1* fusion, *BRAF* mutation, *MET* exon 14 skipping, and PD‐L1 expression) is recommended for patients with advanced or recurrent nonsquamous NSCLC in Japan.^2^ The *EGFR* mutation positivity rate, evaluated using surgical tissues, was 40.1% (352/876) in a prior study of patients with surgically resected stage I–IV NSCLC.^27^ In another cohort study, *EGFR* gene mutations were detected in 49.4% (196/397) of NSCLC patients who underwent surgery, of which 35.2% (69/196) had stage II–IV NSCLC.^28^ Although these studies cannot be directly compared because patients were classified by pathologic stage whereas we used clinical stage, those findings may increase awareness of the need for testing *EGFR* status, especially because *EGFR* gene mutations in patients with lung cancer tend to be more common in Asian than in non‐Asian patients.^29^ Currently several studies are ongoing to evaluate the efficacy of perioperative EGFR‐targeted therapies in patients with resected or resectable NSCLC (e.g., NCT04351555 and NCT03433469; www.clinicaltrials.gov). Once those studies are completed, we would anticipate that the frequency of *EGFR* testing will increase and more accurate data on the prevalence of *EGFR* mutations in patients with stage III NSCLC will become available.

We revealed the outcomes of this cohort of Japanese patients with stage III NSCLC separately for those who underwent surgery alone and those who underwent surgery+perioperative therapy. The median and 3‐year OS, PFS, and DFS rates were favorable in patients who underwent perioperative therapy (not reached, 61.1%, 42.4%, and 47.1%, respectively), and were numerically greatest in those who received both neoadjuvant and adjuvant therapies. However, we should consider that patients who underwent perioperative therapy tended to be younger than those who underwent surgery alone, and the former may be able to tolerate perioperative therapies better.

Since there were some differences in patient characteristics between the surgery and surgery+perioperative therapy groups, it is possible that these differences may influence or confound the prognosis. Therefore, we performed post‐hoc multivariable Cox regression of clinically important confounders to account for the variation in patient characteristics. These analyses showed that perioperative therapy was associated with more favorable OS, PFS, and DFS compared with surgery alone (Figure 3). Although the criteria for perioperative therapy vary widely and the specific treatment modality was not redefined in this study, these results suggest that physicians should consider perioperative therapy for patients who are deemed eligible for such treatments. The analysis also suggested that non‐SCC was associated with more favorable OS compared with SCC. This finding is consistent with the results of prior research in which SCC was associated with worse prognosis than non‐SCC.^30^

We also observed some differences in the type of recurrence as local progression tended to be more common in patients who underwent surgery alone, whereas distant metastasis (with or without local progression) tended to be more frequent in those who underwent surgery+perioperative therapy. This might be due to the patient's background and the reasons for choosing perioperative treatment. We suspect that patients who were more easily controlled locally may have been preferentially selected for surgery, whereas patients considered at higher risk of metastasis may have been selected for perioperative therapy. Although the surgery+perioperative therapy group showed more favorable prognosis, this difference in pattern of recurrence, especially brain metastasis, is an important finding that requires clinical attention. Due to the imbalance in patients and possibility of confounding, further investigations into these findings are needed.

Japanese guidelines suggest the usefulness of adjuvant chemotherapy, particularly cisplatin‐based regimens, based on the results of the IALT, JBR.10, and ANITA studies.^31^, ^32^, ^33^ In other countries, the median OS values for surgery alone in patients with stage III NSCLC were 27 months in Canada^19^ and 24.3 months in the US,^34^ which were comparable to the value (29.3 months) of our study, but were lower than those of patients who underwent surgery+perioperative therapy. These data suggest the survival benefit of perioperative therapy. As a consequence of this evolving treatment strategy that places greater emphasis on perioperative therapies,^35^ it is important that a multidisciplinary team of specialists contribute to the management of patients with stage IIIA NSCLC.^36^, ^37^ This multidisciplinary approach will become a central aspect of patient management once immunotherapies are incorporated into the treatment of resectable stage III (or earlier) NSCLC.^3^

This study was conducted retrospectively and involved a limited number of participating sites (11). Further expansion of the cohort or extending the follow‐up time is not possible. Patients were identified based on the recorded diagnosis of clinical stage III NSCLC, and it is unclear when the physician selected the treatment regimen; for example, we cannot confirm whether the physician selected the postoperative regimen based on preoperative information or whether the pathological diagnosis resulted in changes to the planned treatment. Other limitations include the unequal numbers of patients, not considering causal relationships, and unmeasured confounding, all of which might introduce bias. To mitigate the limitations, we sought to reduce selection bias by performing continuous enrollment. We also performed multivariable analyses of OS, PFS, and DFS, but residual confounding may remain. Nevertheless, the results captured here should provide insight into the real‐world management and outcomes of resectable clinical stage III NSCLC in Japan. Larger studies capturing more detailed clinical data would be helpful to confirm our findings.

In conclusion, perioperative therapy, especially a combination of neoadjuvant+adjuvant therapy, was associated with more favorable OS and PFS. Multivariable analysis also showed that surgery+perioperative therapy was independently associated with more favorable OS, PFS, and DFS compared with surgery alone. Therefore, we believe that perioperative therapy should be considered for patients who are deemed eligible for such therapy in clinical practice. Our findings suggest that aggressive multimodal therapy is appropriate for selected patients with resectable clinical stage III NSCLC, and this regimen could be offered to patients expected to tolerate it.

## AUTHOR CONTRIBUTIONS

Masahiro Tsuboi: Conceptualization, formal analysis, methodology, supervision, visualization, writing–original draft, and writing–review and editing. Haruyasu Murakami: Conceptualization, data curation, formal analysis, investigation, methodology, supervision, visualization, writing–original draft, and writing–review and editing. Hideyuki Harada: Conceptualization, data curation, formal analysis, investigation, methodology, supervision, visualization, writing–original draft, and writing–review and editing. Tomotaka Sobue: Conceptualization, formal analysis, methodology, supervision, visualization, writing–original draft, and writing–review and editing. Tomohiro Kato: Conceptualization, data curation, investigation, visualization, and writing–review and editing. Shinji Atagi: Conceptualization, data curation, investigation, visualization, and writing–review and editing. Takaaki Tokito: Conceptualization, data curation, investigation, visualization, and writing–review and editing. Tadashi Mio: Conceptualization, data curation, investigation, visualization, and writing–review and editing. Hirofumi Adachi: Conceptualization, data curation, investigation, visualization, and writing–review and editing. Toshiyuki Kozuki: Conceptualization, data curation, investigation, visualization, and writing–review and editing. Takashi Sone: Conceptualization, data curation, investigation, visualization, and writing–review and editing. Masahiro Seike: Conceptualization, data curation, investigation, visualization, and writing–review and editing. Shinichi Toyooka: Conceptualization, data curation, investigation, visualization, and writing–review and editing. Hiroshi Kitagawa: Conceptualization, formal analysis, funding acquisition, methodology, project administration, resources, software, supervision, validation, visualization, writing–original draft, and writing–review and editing. Ryo Koto: Conceptualization, formal analysis, funding acquisition, methodology, project administration, resources, software, supervision, validation, visualization, writing–original draft, and writing–review and editing. Satoshi Yamazaki: Conceptualization, formal analysis, funding acquisition, methodology, project administration, resources, software, supervision, validation, visualization, writing–original draft, and writing–review and editing. Hidehito Horinouchi: Conceptualization, data curation, formal analysis, investigation, methodology, supervision, visualization, writing–original draft, and writing–review and editing.

## FUNDING INFORMATION

This study was funded by AstraZeneca K.K.

## CONFLICT OF INTEREST STATEMENT

Masahiro Tsuboi received research grants from Nippon Boehringer Ingelheim, MSD, AstraZeneca, Ono Pharmaceutical, Bristol‐Myers Squibb, Eli Lilly Japan, and Novartis; personal and lecture fees from Johnson & Johnson Japan, AstraZeneca, Eli Lilly Japan, Chugai Pharmaceutical, MSD, Bristol‐Myers Squibb, Teijin Pharma, Taiho Pharmaceutical, and Ono Pharmaceutical; personal fees from Medtronic Japan; has participated in a data safety monitoring board for Chugai Pharmaceutical; and has participated in advisory boards for AstraZeneca, MSD, and Novartis. Haruyasu Murakami has received support in relation to this manuscript from AstraZeneca; research grants from AstraZeneca, Takeda Pharmaceutical, Daiichi Sankyo, AbbVie, and IQVIA; and honoraria from AstraZeneca, Chugai Pharmaceutical, Takeda Pharmaceutical, Daiichi Sankyo, Ono Pharmaceutical, Bristol‐Myers Squibb, MSD, Pfizer, Novartis, Eli Lilly Japan, and Taiho Pharmaceutical; and participated on an advisory board for Chugai Pharmaceutical. Hideyuki Harada received support for this manuscript from AstraZeneca, and has received lecture fees from Hitachi, AstraZeneca, Brainlab, Accuray, Chugai Pharmaceutical, Eisai, Taiho Pharmaceutical, Takeda Pharmaceutical, Pfizer, MSD, and Nihon Medi‐Physics. Tomohiro Kato has received honoraria from AstraZeneca, Chugai Pharmaceutical, Kyowa Kirin, Eli Lilly, MSD, Boehringer Ingelheim, Taiho Pharmaceutical, Ono Pharmaceutical, Nippon Kayaku, Takeda Pharmaceutical, and Novartis. Shinji Atagi received support for this manuscript from AstraZeneca, and research grants from AstraZeneca, Eli Lilly, Ono Pharmaceutical, Taiho Pharmaceutical, Boehringer Ingelheim, Pfizer, Bristol‐Myers Squibb, MSD, Chugai Pharmaceutical, Merck, and F. Hoffmann‐La Roche. Takaaki Tokito has received lecture fees from Chugai Pharmaceutical, AstraZeneca, MSD, Novartis, and Bristol‐Myers Squibb. Toshiyuki Kozuki has received research grants from Chugai Pharmaceutical, AstraZeneca, Eli Lilly Japan, Taiho Pharmaceutical, Bristol‐Myers Squibb, Ono Pharmaceutical, MSD, Kyowa Kirin, Merck Biopharma, Daiichi Sankyo, Abbvie, Amgen, Sanofi, Eisai, and Labcorp Development Japan, and personal fees from Chugai Pharmaceutical, AstraZeneca, Eli Lilly Japan, Taiho Pharmaceutical, Bristol‐Myers Squibb, Ono Pharmaceutical, MSD, Pfizer Japan, Kyowa Kirin, Merck Biopharma, Nippon Boehringer Ingelheim, Nippon Kayaku, Novartis, Daiichi Sankyo, Takeda Pharmaceutical, AbbVie, Bayer, and Sawai. Masahiro Seike has received lecture fees from AstraZeneca, MSD, Chugai Pharmaceutical, Taiho Pharmaceutical, Nippon Kayaku, Ono Pharmaceutical, Bristol‐Myers Squibb, Eli Lilly Japan, Takeda Pharmaceutical, Kyowa Kirin, and Novartis, and scholarship donations from Taiho Pharmaceutical, MSD, Chugai Pharmaceutical, Eli Lilly Japan, Nippon Kayaku, and Nippon Boehringer Ingelheim. Hiroshi Kitagawa and Ryo Koto are employees of AstraZeneca. Satoshi Yamazaki was an employee of AstraZeneca at the time of the study. Hidehito Horinouchi has received research funds from MSD, AbbVie, AstraZeneca, Bristol‐Myers Squibb, Ono Pharmaceutical, Merck Biopharma, Daiichi Sankyo, Janssen, Genomic Health, Chugai Pharmaceutical, Roche, and Novartis; honoraria from AstraZeneca, MSD, Eli Lilly, Ono Pharmaceutical, Bristol‐Myers Squibb, Chugai Pharmaceutical, Roche, Kyowa Kirin, and Novartis; and has participated on advisory boards for AstraZeneca, Eli Lilly, Chugai Pharmaceutical, Roche, Ono Pharmaceutical, Bristol‐Myers Squibb, and MSD. Tomotaka Sobue, Tadashi Mio, Hirofumi Adachi, Takashi Sone, and Shinichi Toyooka have no conflicts of interest to declare.

## Supporting information


**TABLE S1.** *EGFR* mutation status by first‐line treatment and histological type.
**TABLE S2.** Perioperative treatments.
**TABLE S3.** Number of deaths within 30 or 90 days according to first‐line treatment.
**FIGURE S1.** (a) Study design. (b) Patient disposition.
**FIGURE S2.** Disease‐free survival in (a) the overall surgery cohort, (b) patients who underwent surgery alone or surgery+perioperative therapy, and (c) according to the type of perioperative therapy.
**FIGURE S3.** Cumulative incidence of distant metastasis in patients who underwent surgery alone or surgery+perioperative therapy.

## Data Availability

Data underlying the findings described in this study may be obtained in accordance with AstraZeneca's data sharing policy described at https://astrazenecagrouptrials.pharmacm.com/ST/Submission/Disclosure.
