# Supplementary material for: Treatment patterns and clinical outcomes of resectable clinical stage III non‐small cell lung cancer in a Japanese real‐world setting: Surgery cohort analysis of the SOLUTION study
Source: Thorac Cancer. 2024 May 29;15(20):1541–52. doi: 10.1111/1759-7714.15305 (PMC11246785; doi:10.1111/1759-7714.15305)
Supplement: Supplementary file 1 — TABLE S1. EGFR mutation status by first‐line treatment and histological type. TABLE S2. Perioperative treatments. TABLE S3. Number of deaths within 30 or 90 days according to first‐line treatment. FIGURE S1. (a) Study design. (b) Patient disposition. FIGURE S2. Disease‐free survival in (a) the overall surgery cohort, (b) patients who underwent surgery alone or surgery+perioperative therapy, and (c) according to the type of perioperative therapy. FIGURE S3. Cumulative incidence of distant metastasis in patients who underwent surgery alone or surgery+perioperative therapy. [file TCA-15-1541-s001.pdf]

SUPPLEMENTARY MATERIAL

Treatment patterns and clinical outcomes of resectable clinical stage III non-small cell lung cancer in a Japanese real-world setting: surgery cohort analysis of the SOLUTION study

Masahiro Tsuboi, Haruyasu Murakami, Hideyuki Harada, Tomotaka Sobue, Tomohiro Kato, Shinji Atagi, Takaaki Tokito, Tadashi Mio, Hirofumi Adachi, Toshiyuki Kozuki, Takashi Sone, Masahiro Seike, Shinichi Toyooka, Hiroshi Kitagawa, Ryo Koto, Satoshi Yamazaki, and Hidehito Horinouchi

**Contents**	**Page**
**TABLE S1.** *EGFR* mutation status by first-line treatment and histological type	2
**TABLE S2.** Perioperative treatments	3
**TABLE S3** Number of deaths within 30 or 90 days according to first-line treatment	5
**FIGURE S1.** (a) Study design. (b) Patient disposition	6
**FIGURE S2.** Disease-free survival in (a) the overall surgery cohort, (b) patients who underwent surgery alone or surgery+perioperative therapy, and (c) according to the type of perioperative therapy	7
**FIGURE S3.** Cumulative incidence of distant metastasis in patients who underwent surgery alone or surgery+perioperative therapy	10

TABLE S1. *EGFR* mutation status by first-line treatment and histological type

		Tested before first-line treatment	*EGFR* mutation status [a]		
			Positive	Negative	Unknown
Overall surgery cohort (n = 149)	Adenocarcinoma (n = 70)	38 (54.3)	6 (15.8)	31 (81.6)	1 (2.6)
	SCC (n = 61)	9 (14.8)	0	9 (100.0)	0
	NET (NSCLC) (n = 4)	0	n/a	n/a	n/a
	Other (n = 14)	5 (35.7)	0	5 (100.0)	0
Surgery alone (n = 67)	Adenocarcinoma (n = 25)	9 (36.0)	0	8 (88.9)	1 (11.1)
	SCC (n = 32)	4 (12.5)	0	4 (100.0)	0
	NET (NSCLC) (n = 2)	0	n/a	n/a	n/a
	Other (n = 8)	2 (25.0)	0	2 (100.0)	0
Surgery+perioperative therapy (n = 82)	Adenocarcinoma (n = 45)	29 (64.4)	6 (20.7)	23 (79.3)	0
	SCC (n = 29)	5 (17.2)	0	5 (100.0)	0
	NET (NSCLC) (n = 2)	0	n/a	n/a	n/a
	Other (n = 6)	3 (50.0)	0	3 (100.0)	0

Note: Values are *n* (%).

Abbreviations: EGFR, epidermal growth factor receptor; NET, neuroendocrine tumor; NSCLC, non-small cell lung cancer; SCC, squamous cell carcinoma.

[a] Percentages were calculated using the number of patients who underwent testing prior to first-line treatment as the denominator.

TABLE S2. Perioperative treatments

**Neoadjuvant+adjuvant therapies (*n* = 17)**		**Neoadjuvant therapies (*n* = 24)**		**Adjuvant therapies (*n* = 41)**	
**Regimen**	***n* (%)**	**Regimen**	***n* (%)**	**Regimen**	***n* (%)**
Neoadjuvant therapy	17 (100.0)	Neoadjuvant therapy	24 (100.0)	Adjuvant therapy	41 (100.0)
RT alone	0	RT alone	1 (4.2)	RT alone	2 (4.9)
CRT [a]	12 (70.5)	CRT [a]	17 (70.8)	CRT [a]	2 (4.9)
Cisplatin+docetaxel	10 (83.3)	Cisplatin+docetaxel	13 (76.5)	Cisplatin+vinorelbine	1 (50.0)
Cisplatin+vinorelbine	2 (16.7)	Cisplatin+vinorelbine	3 (17.6)	Cisplatin+S-1	1 (50.0)
CT alone [a,b]	5 (29.4)	Cisplatin+paclitaxel	1 (5.9)	CT alone [a]	37 (90.2)
Carboplatin+nab-paclitaxel	2 (40.0)	CT alone [a,b]	6 (25.0)	Cisplatin+vinorelbine	16 (43.2)
Cisplatin+pemetrexed	2 (40.0)	Carboplatin+paclitaxel	3 (50.0)	Cisplatin+S-1	3 (8.1)
Carboplatin+pemetrexed	1 (20.0)	Carboplatin+pemetrexed	2 (33.3)	Cisplatin+pemetrexed	3 (8.1)
Cisplatin+vinorelbine	1 (20.0)	Cisplatin+pemetrexed	2 (33.3)	Cisplatin+irinotecan	2 (5.4)
Adjuvant therapy	17 (100.0)			Carboplatin+paclitaxel	2 (5.4)
RT alone	1 (5.9)			UFT	2 (5.4)
CRT	0			Cisplatin+pemetrexed+anti-VEGF	1 (2.7)
CT alone [a]	16 (94.1)			Cisplatin+etoposide	1 (2.7)
Cisplatin+docetaxel	9 (56.3)			Carboplatin+nab-paclitaxel	1 (2.7)
Carboplatin+nab-paclitaxel	2 (12.5)			Carboplatin+pemetrexed	1 (2.7)
Cisplatin+pemetrexed	1 (6.3)			Carboplatin+gemcitabine	1 (2.7)
Cisplatin+pemetrexed+anti-VEGF	1 (6.3)			S-1	1 (2.7)
Carboplatin+pemetrexed	1 (6.3)			Other	1 (2.7)

Neoadjuvant+adjuvant therapies (*n* = 17)		Neoadjuvant therapies (*n* = 24)		Adjuvant therapies (*n* = 41)	
**Regimen**	***n* (%)**	**Regimen**	***n* (%)**	**Regimen**	***n* (%)**
Cisplatin+vinorelbine	1 (6.3)			Missing	2 (5.4)
Docetaxel	1 (6.3)				

Note: Values are *n* (%). Some patients received multiple therapies. If the same patient received the same therapy multiple times, the patient was counted once in that category.

Abbreviations: RT, radiotherapy; CRT, chemoradiotherapy; CT, chemotherapy; VEGF, vascular endothelial growth factor; UFT, uracil-tegafur.

[a] The percentages of patients who received individual CRT or CT regimens were calculated using the number of patients who received CT or CRT as the denominator.

[b] One patient received two different treatment regimens.

TABLE S3. Number of deaths within 30 or 90 days according to first-line treatment

Time from primary surgery	Overall surgery cohort (*n* = 149)	Surgery alone (*n* = 67)	Surgery+ perioperative therapy (*n* = 82)
≤30 days	1 (0.7)	1 (1.5)	0
≤90 days	2 (1.3)	2 (3.0)	0

Note: Values are n (%) of patients who died.

FIGURE S1. (a) Study design. (b) Patient disposition.

CRT, chemoradiotherapy; CT, chemotherapy; NSCLC, non-small cell lung cancer; RT, radiotherapy.

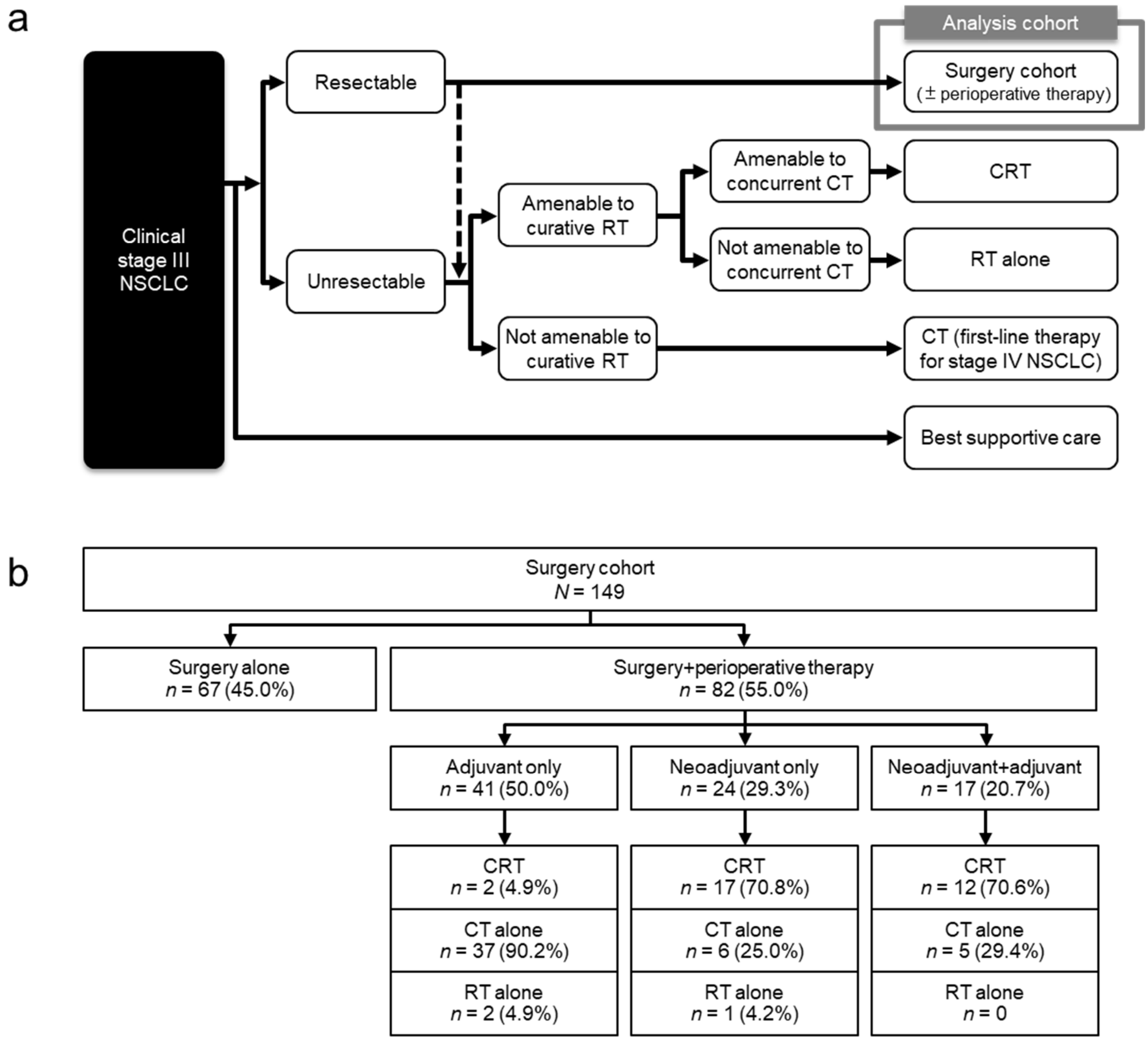

FIGURE S2. Disease-free survival in (a) the overall surgery cohort, (b) patients who underwent surgery alone or surgery+perioperative therapy, and (c) according to the type of perioperative therapy.

adju, adjuvant therapy; CI, confidence interval; DFS, disease-free survival; mo, month; neoadju, neoadjuvant therapy; NR, not reached; yr, year.

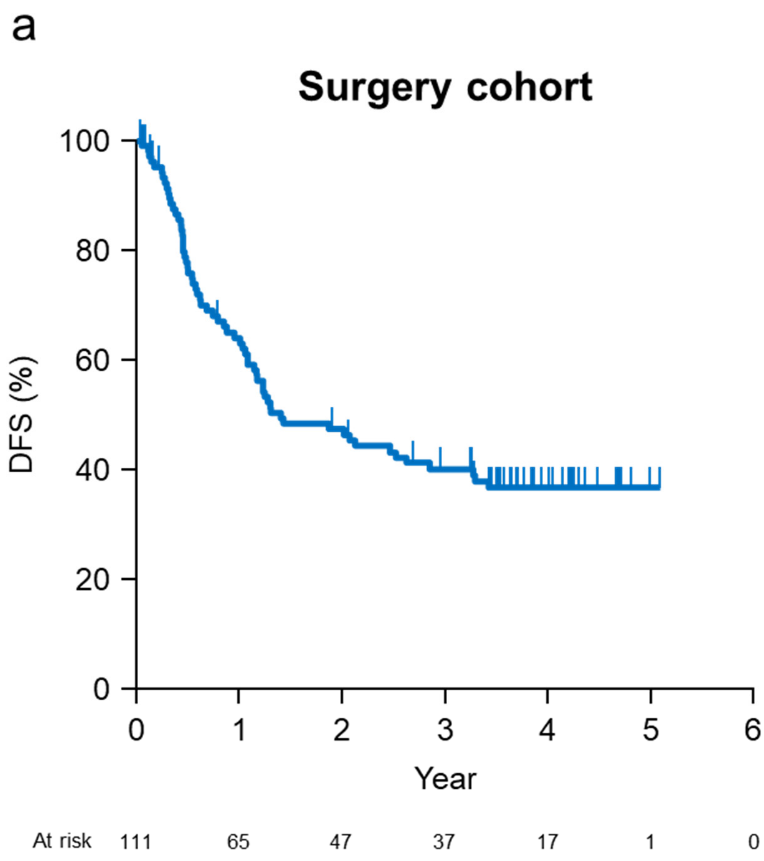

	n	Median DFS, mo (95% CI)	DFS at 2 yr (95% CI)	DFS at 3 yr (95% CI)	DFS at 5 yr (95% CI)
Surgery cohort	111	16.7 (12.9–34.1)	47.4% (37.4%–56.6%)	40.2% (30.6%–49.5%)	36.7% (27.3%–46.1%)

b

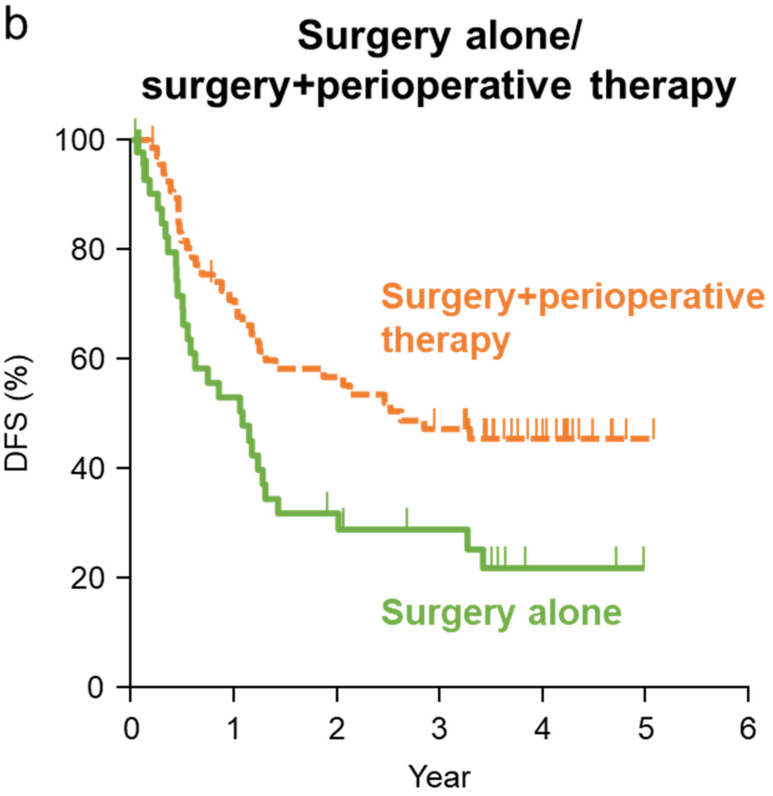

At risk							
Surgery alone	45	20	11	8	2	0	0
Surgery+ perioperative	66	45	36	29	15	1	0

	n	**Median DFS, mo (95% CI)**	**DFS at 2 yr (95% CI)**	**DFS at 3 yr (95% CI)**	**DFS at 5 yr (95% CI)**
Surgery alone	45	12.9 (5.9–15.5)	31.8% (17.9%–46.6%)	28.9% (15.6%–43.7%)	-
Surgery+ perioperative	66	31.5 (14.7–NR)	56.6% (43.6%–67.6%)	47.1% (34.6%–58.7%)	45.4% (32.9%–57.1%)

C

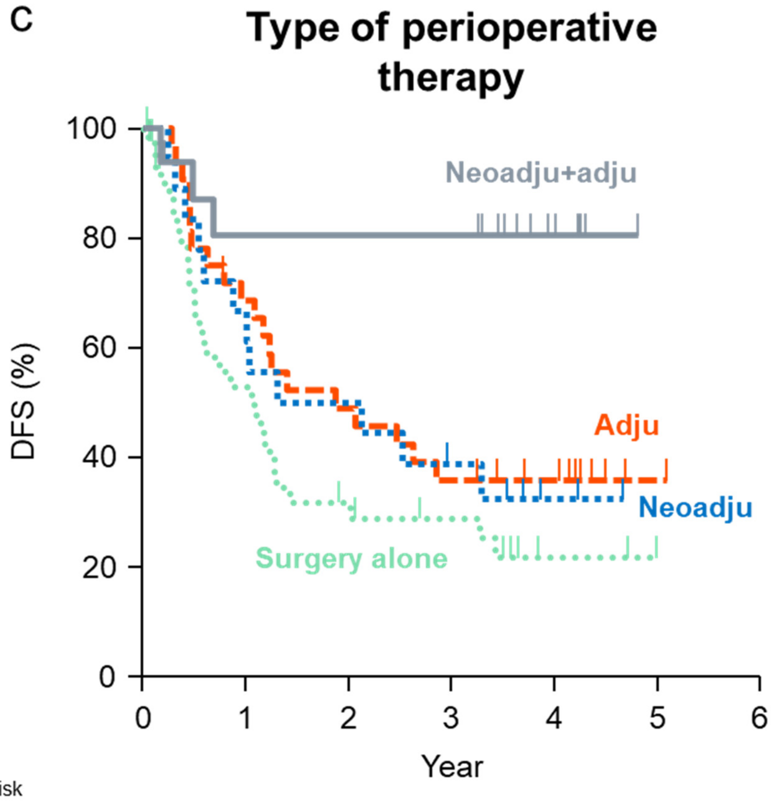

At risk							
Neoadju+adju	16	12	12	12	5	0	0
Neoadju	18	12	9	6	2	0	0
Adju	32	21	15	11	8	1	0
Surgery alone	45	20	11	8	2	0	0

	n	**Median DFS, mo (95% CI)**	**DFS at 2 yr (95% CI)**	**DFS at 3 yr (95% CI)**	**DFS at 5 yr (95% CI)**
Neoadju+ adju	16	NR (NR–NR)	80.4% (50.6%–93.2%)	80.4% (50.6%–93.2%)	-
Neoadju only	18	20.5 (7.0–NR)	50.0% (25.9%–70.1%)	38.9% (17.5%–60.0%)	-
Adju only	32	22.4 (11.3–NR)	49.0% (30.8%–65.0%)	35.9% (19.8%–52.9%)	35.9% (19.8%–52.4%)
Surgery alone	45	12.9 (5.9–15.5)	31.8% (17.9%–46.6%)	28.9% (15.6%–43.7%)	-

No

FIGURE S3. Cumulative incidence of distant metastasis in patients who underwent surgery alone or surgery+perioperative therapy.

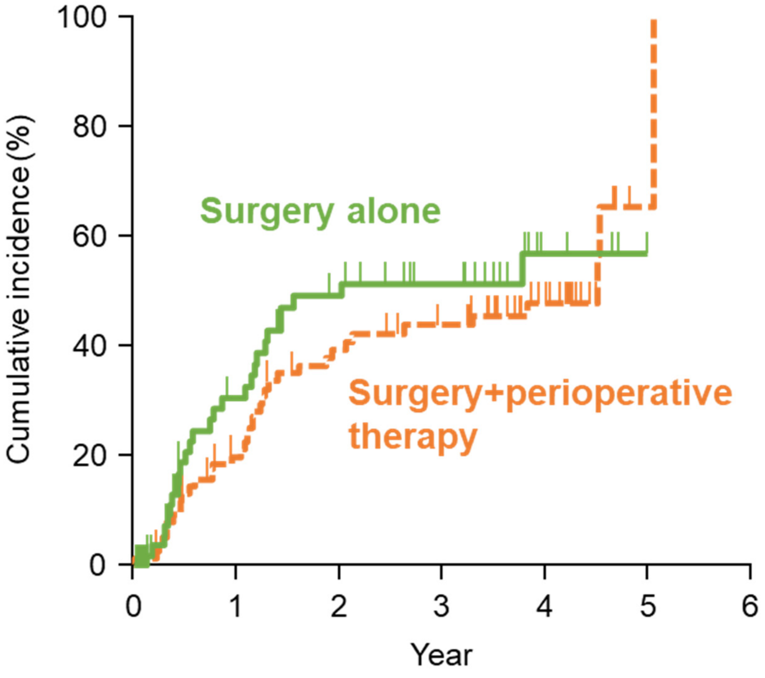

At risk							
Surgery alone	66	34	23	16	4	0	0
Surgery+ perioperative	78	58	42	36	19	1	0
